# Mental health in children with and without disabilities in a register-based Swedish sample supports the two-continua model: a latent class analysis

**DOI:** 10.1186/s12889-025-23388-7

**Published:** 2025-06-19

**Authors:** Lina Homman, Lilly Augustine, Mats Granlund

**Affiliations:** 1 Disability research division, Institution of behaviour and learning, Linköping university, Linköping, Sweden; 2 CHILD, School of Education and Communication, Jönköping university, Jönköping, Sweden; 3 CHILD, School of health and welfare, Jönköping university, Jönköping, Sweden

**Keywords:** Mental health, Disabilities, Children, Latent class analysis, Two-continua model

## Abstract

**Background:**

Mental health is a term frequently used to describe mental health problems. However, mental health includes both mental health problems and well-being. Therefore, mental health can be seen as having two distinct yet related dimensions, as described in the two-continua model of mental health (Westerhof & Keyes, 2010) where an individual can simultaneously experience any combination of well-being and problems, suggesting four classes: (i) high well-being, low problems; (ii) high well-being, high problems; (iii) low well-being, low problems; and (iv) low well-being, high problems. Through this framework an understanding of differences in putative risk and protective factors can be gained when compared across classes. While the model has received support, it is unclear how it applies to children. In particular, children with disabilities, as disabilities pose a risk factor to poor mental health. A greater understanding of similarities and differences between children with and without disabilities, and of risk and protective factors, could help tailor support focused on enhancing well-being, both as a goal and as a means to better self-management of mental health.

**Methods:**

The present project utilizes Sweden Statistics (SCB) study (barnULF) to measure life conditions. Nearly 4000 children aged 10–18, with and without disabilities, and their caregivers (ULF/SILC) were studied through yearly interview-based sample surveys conducted between 2013 and 2019. Latent class analysis was performed to assess whether the data fit a 4-class model in line with the two-continua model. Possible factors influencing mental health, including participation, were compared across the identified classes and between children with and without disabilities.

**Results:**

The analysis confirmed the predicted model. Each class showed distinct features regarding putative risk and protective factors of mental health and demographics in both the child and caregiver. These features differed significantly between children with and without disabilities, especially relating to participation, social bonds, family functioning, digital media use, and perceived safety. Age, disability, and gender predicted class adversity.

**Conclusions:**

The results suggest that mental health problems and well-being are two related but separate constructs, highlighting the importance of promoting participation and recognizing well-being and not just mental health problems when planning interventions. The results also highlight the importance of providing support for not only the child but also their caregiver.

## Background

In 1948 WHO stated that health is “*a state of complete physical*,* mental*,* and social well-being and not merely the absence of disease or infirmity*” [1]. This definition states that well-being is a multidimensional construct including positive experiences. Yet traditionally, mental health has been considered as the absence of mental health problems, focusing on a treatment paradigm with mental health as a continuum from illness to wellness defined only as the lack of symptoms of problems [2, 3]. A difficulty in using a unidimensional definition focused on mental health problems to define mental health, is that the degree of wellness as a phenomenon of its own is not visible [4]. Therefore, based on Jahoda’s work [3], Westerhof and Keyes [5] have suggested a two-continua model where well-being is seen as a second continuum separate from mental health problems. Here, the two-continua are distinct but related and are partly independent of each other. This two-continuum suggests poor mental health (languishing) (low well-being and high mental health problems) at the negative end of the continuum and good mental health (flourishing) (high well-being and low mental health problems) at the positive end. In addition to these two groups, Westerhof and Keyes also identify two intermediate groups with different levels of well-being and mental health problems.

In their study, mental health problems were assessed as ranging from a diagnosed mental health condition to mild mental health problems, measured with traditional mental health questionnaires, while well-being was measured with the Mental Health Continuum-Short Form (MHC-SF). The MHC-SF was based on the assumption that mental well-being has three, only partly overlapping dimensions: emotional (subjective) well-being, psychological well-being and social well-being [6]. Criticism towards these three dimensions has been raised, arguing that this is just one dimension [7, 8]. Arguably mental health constitutes of more aspects than just emotions, situations are appraised, and people participate to different extent, indicating more than unidimensionality, if so this ought to be visible using other questionnaires as well.

The notion that mental well-being is partly independent from mental health problems has received support amongst healthy adults [5], adults with mental health diagnosis [9], and adults with autism [10]. However, studies are lacking on whether the two-continua model is applicable to children, who differ from adults in several aspects such as social and biological as well regarding mental health in both epidemiology and symptoms presentation [11]. It is therefore unclear whether the results are transferable to children, which is of high relevance as mental health has become of major concern in youth due to its increase in prevalence over the past decades [12, 13], highlighting the importance of a good knowledge base in order to promote well-being and prevent mental health problems. In addition, some children are at increased risk of poor mental health, for example children with disabilities [14], as disabilities, and the social construction of disabilities, forms a hinder to factors which promote mental well-being and protects against mental health problems, factors such as participation, suggesting it is of importance to gain better knowledge on mental health amongst children with disabilities through the lens of the dual-continua model as this model may provide additional explanatory power.

### Mental health in children

In children, well-being is related to the achievement of psychological, social and emotional milestones [15], while mental health problems include changes in emotions, thinking or behaviour associated with distress and difficulties functioning in normal daily activities. Mental health problems in childhood most commonly include internalising disorders, such as anxiety and depression, and externalising disorders, such as conduct disorders. They are often partly a consequence of life events [16]. Mental health problems peak during adolescence, followed by a decrease and stabilisation in adulthood [16, 17]. The increase during this time is often attributed to maturation and changes of the adolescent, such as physical, hormonal, cognitive, social, legal, psychological, and behavioural attributes [18, 19]. Known environmental risk factors of mental health problems for typically developed children include several school-, peers-, afterschool-, and family factors such as being the victim of bullying [20], the quality of peer relationships [21], socioeconomic status of the family [22], prevalence of mental or physical illness amongst caregivers [23], different forms of neglect and abuse [24], low participation in afterschool activities such as physical exercise [25], parental conflict or family breakdown, large family size, early parenthood, and overcrowding in the home [26]. The current increase in poor mental health has been attributed to a range of factors such as increased academic pressure, climate change, and social media usage [13, 27, 28].

### Mental health in children with disabilities

A group of individuals at particular risk of poor mental health are children with disabilities, not least those of a neurodevelopmental nature, as they have an on average higher levels of mental health problems and mental illness and lower level of mental health well-being in comparison to their peers without disabilities [14, 29, 30]. While several of these studies are focused on children with a particular diagnosis e.g. ADHD or ASD, it is probable that many children with different Neuro Developmental Disorder (NDD) [31] and other diagnoses related to problems with cognition and emotional regulation, e.g. cerebral palsy have partly the same problems with everyday functioning [4, 32] and thus may need the similar or related interventions.

Many studies do indicate that mental health outcomes are adversely affected by disability status [33, 34] and the possible psychosocial disadvantages of disabilities [29] indicate that children with disabilities are more likely to be exposed to a higher degree of contextual disadvantage [35] and of certain risk factors as compared to children of typical development, such as bullying [36], victimisation [35], and difficulties with participation in physical and social activities, which seem to have strong predictive value. However, there is no exact explanation for the differences between children and adolescents with disabilities versus those without and certain risk factors of poor mental health are the same in children with and without disabilities, such as low family socioeconomical status and mothers’ educational level as indicated by a systematic review of longitudinal trajectories of mental health problems in children and youth with disabilities [37].

Preventative measures on individual level cannot always tackle structural risk factors, which is why identifying protective factors of mental health problems and factors promoting well-being are essential [38]. Concerning positive experiences, evidence indicate protective factors such as personal resources (e.g., optimism, self-efficacy [39, 40]), familial resources (e.g., parental support, parenting style [41]), social resources (e.g., relationships outside the family [42]) and being part of families oriented towards social-recreational activities; all factors which vary in impact with age and gender [14, 43].

Participation, in particular within community activities, is highlighted as an important protective factor of well-being (e.g [14, 44]). with family factors explaining more than disability on frequency of participation. However, individuals with disabilities do face more participatory restrictions [45] and participate less in meaningful activities and in school than peers of typical development [14, 45–47]. The fact that individuals with disabilities report spending too much time in sedentary activities [48] and report increased perceived loneliness [49], stresses the importance of promoting participation in life activities as protective factors for well-being. According to a longitudinal study by Augustine [14] the positive relation between well-being and participation is stronger than the negative relation between mental health problems and participation, indicating that enhancing people’s participation in life situations may lead to well-being and facilitate self-management of mental health problems, supporting the notion of a two-continua model. In promoting and applying effective interventions for mental health amongst children with disabilities, knowledge about the relationships between well-being, mental health problems and other impacting factors in a child’s life is necessary to understand the mechanisms of enhancing mental health. However, there is a lack of knowledge regarding the interplay of these factors from a two-continua perspective in children with and without disabilities.

### Aim

In order to improve mental health amongst children, with and without disabilities, a good knowledge base is necessary. To compare the two groups is essential as interventions and preventative measures too often are based on typically developed children and then applied to all children. To only work with mental health problems fails to account for mental wellbeing and the nuances of the two concepts combined. The dual-continua model offers a framework where mental health can be perceived and worked with through a nuanced perspective and where associated factors can be compared across classes. However, the literature is lacking regarding the two-continua model amongst children. Studies which have found support for the two-continua model e.g., Eriksson & Stattin [50, 51] used a person-based factor analysis to create four groups of mental health profiles based on the two-continua model and identified a model with good construct validity: four groups with distinct features among 11–15-year-olds; however, they did not assess children with disabilities. Täljedal and colleagues [52] did, using a cluster analysis on a smaller sample of children (*n* = 136), and noted that that patterns of mental health problems and well-being amongst children do co-exist amongst children with disabilities. However, they did not assess data in line with the dual-continua model as they assessed items from the Strength and Difficulties Questionnaire (SDQ) (conduct, emotional, prosocial behaviours) [53]. Nevertheless, extended knowledge is needed on children and children with disabilities and how they differ from one another in mental health and risk and protective factors of menta health (if they do) through the lens of the two-continua model. Therefore, the aim of the present study was twofold. First, to assess the feasibility of the two-continua model on a sample of children, with and without disabilities, through a person-centred analysis to create classes of individuals sharing similar profiles of mental health as a construct of 4 classes (varying in high/low mental health problems/well-being) independent of disability status. Followed by an assessment of whether it was more or less likely for children with disabilities to be in particular classes as compared to children without disabilities. Secondly, to investigate whether putative risk and protective factors, including participation, of both the child and caregivers, differed across classes and between children with and without disability.

## Methods

### Study design and data collection

The study utilises Sweden Statistics (SCB) register on the conditions of *Living Conditions Survey of Children* (barnULF) [54], and their caregivers (ULF/SILC (Statistics on Income and Living Conditions, ULF in Swedish)) [55], interview-based sample surveys undertaken yearly in a repeated cross-sectional design where recruitment is done through a randomized selection of the Swedish population. ULF/SILC consists of structured telephone interview-based data of individuals aged 16 years and older, concerning their household, income, work, health, partners, and children, and linked to relevant registry-based data. Consent to participation was provided verbally at the beginning of the interview, the interview was recorded and deleted within 3 months. If the participant in ULF/SILC had a child in the household above the age of 10/12, they were asked whether they would consent for the child to participate in barnULF, both caregivers (or one caregiver if the child only lived with one caregiver) had to provide consent for the child to participate. If consent for the child’s participation was provided, the child was provided with information about the study and the interviewer confirmed the child had understood, followed by inclusion of the child in the study if they so wished. Each year 12,000–13,000 individuals are interviewed, whereof 1000–1500 are children of whom 200–300 have a disability which impacts their daily life. The information from ULF/SILC and barnULF is linked prior to de-identification. Inclusion criteria for barnULF are age (10–18 years of age in 2013 and 12–18 year of age from 2014 onwards) and residence of the child (must live at a minimum of 50% in the same accommodation as the adult participant of ULF/SILC). The data collection is in line with GDPR standards, and the present study has received ethical approval from the Swedish Ethical board (Etikprövningsnämnden).

### Participants

Children from 2013 to 2016 and 2018–2019 were included in the study, resulting in 3682 children. Data on disability was not collected prior to 2013 and was not collected in 2017 whereof data from these years was excluded. Participants were excluded if data were missing on all the mental health items (*n* = 5). One participant had only replied to one mental health item and was also excluded, resulting in a total sample of 3676 participants. Majority of the participants had provided data on all the mental health items (*N* = 3586 (97,55%)), the remaining had missing data on 1 item (*N* = 74, 2.01%), 2 items (*N* = 9, 0.24%) or 3–5 items (*N* = 7, 0.18%). The sample consisted of 1945 girls and 1731 boys with a mean age of 14.41 (SD = 2.29). Information on children’s caregivers was included in the study, resulting in 2804 caregivers. A total of 510 (269 boys and 241 girls) children reported a disability (see Table 1). However, 82 of these children reported more than one of the listed disabilities, a comorbid condition, which is described separately (see comorbid & Comorbid Total* in Table 1) and not accounted for in the listed numbers in Table 1.

**Table 1:** Demographic information of children, by year

	2013 (*n* = 724)	2014 (*n* = 677)	2015 (*n* = 553)	2016 (*n* = 547)	2018 (*n* = 607)	2019 (*n* = 569)	Total (*n* = 3676)	Comorbid Total (*N* = 3676)
Age (Mean (SD))	13.75 (2.56)	13.81 (2.59)	14.87 (2.01)	14.92 (2.00)	14.64 (1.97)	14.88 (2.00)	14.41 (2.29)	
Girls (% (N))	54.14 (392)	52.29 (354)	54.07 (299)	53.75 (294)	49.42 (300)	53.95 (307)	52.92 (1946)	
***Disability (% (N))***								
Hearing impairment	1.25 (9)	0.89 (6)	1.81 (10)	0.73 (4)	0.99 (6)	1.76 (10)	1.22 (45)	0.95 (35)
Visual impairment	1.80 (13)	2.22 (15)	0.54 (3)	0.37 (2)	0.82 (5)	1.58 (9)	1.28 (47)	0.92 (34)
Mobility impairment	0.97 (7)	0.74 (5)	2.53 (14)	1.46 (8)	0.66 (4)	0.88 (5)	1.17 (43)	0.71 (26)
ADHD/ASD	3.18 (23)	2.81 (19)	3.25 (18)	3.66 (20)	5.77 (35)	4.22 (24)	3.78 (139)	2.42 (89)
Dyslexia etc.	5.11 (37)	3.69 (25)	7.96 (44)	6.40 (35)	7.25 (44)	5.80 (33)	5.93 (218)	4.51 (166)
Other	4.28 (31)	1.92 (13)	4.16 (23)	3.47 (19)	2.14 (13)	3.51 (20)	3.24 (119)	2.12 (78)
Comorbid	3.18 (23)	0.89 (6)	2.71 (15)	1.65 (9)	2.64 (16)	2.28 (13)	-	2.23 (82)
Any	16.57 (120)	12.26 (83)	20.25 (112)	16.09 (88)	17.63 (107)	17.75 (101)	13.87 (510)	-

*Total: only including individuals with comorbid conditions in the comorbid group

*Comorbid*: refers to children with more than one disability; *Any*: refers to children with at least one disability

### Material

Children provided information on basic demographics as well as on their mental health (well-being and problems), school, family, friends, and participation. Children’s caregivers provided information on health, mental health, disabilities, financial situation, risk-taking behaviours, occupation, housing, family etc. Caregivers also provided information on whether the child had a disability or not which impacted their daily life. The scales used were not part of pre-existing validated scales measuring these constructs but were based in the literature (e.g [56]). and are in the interviews updated in line with social change (such as social media). Within certain areas, items addressing the same topic were summed as to compress the information as well as to account for variability on the topic. The method used (summarising items) is common in studies using secondary data (e.g [56]). as a pragmatic approach is often necessary. In some instances, this was performed as (a) items were answered as yes or no and (b) focus was not on the particular event but the frequency of the event where a higher score meant a higher frequency (physical participation, cultural participation, household participation, areas of perceived safety, and number of others they could confide in). In other instances, items were answered on a likert scales and summed as each item investigated a specific dimension of a phenomenon, however items did not directly correspond to other established instruments but were summed based on addressing the topic of interest (solitary participation, social participation, digital media use, and parental participation).

#### Disability in the child impacting their daily life

Disabilities asked for on a binary scale were dyslexia/dyscalculia/speech or language impairment, mobility impairment, ADHD/autism/Aspergers (ADHD/ASD), hearing impairment, visual impairment not possible to correct with aids, and other disability. An additional binary disability item was created based on the disability items where children were defined as having a disability or not.

#### Mental health

Twelve items were identified as indicators of mental health problems and well-being. The items measured 9 indicators on mental health problems; poor sleep, headache, stomach-ache, tired in school, trouble falling asleep, stress, feeling down, difficulties concentrating, nervous and tense, and three indicators of well-being; happy with oneself, good mood, and good general well-being. Mental health problem items were chosen based on thematic resemblance to well-established screening tools such as the SDQ and CBCL. The items have been shown to support a latent construct of mental health problems [57]. Some items had been changed somewhat across the years in wording and number of response levels whereof some response levels had to be collapsed to enable item merge across the years. After recoding, items were scored on a 4 or 5 item Likert scale where a score of 1 = absence of mental health item (low problems//low score on well-being) and a score of 4 or 5 = high prevalence of the mental health item (high level of problem//high level of well-being).

#### Participation (attendance)

Physical participation consisted of two items, (i) participation in organised physical activity and (ii) physical activity on own accord; each answered as yes or no (where yes was coded as 1) and summed together (scale of 0–2). Cultural participation consisted of five separate items measuring attendance to the cinema, library, theatre, concert, and museum in the past 6 months; each activity was scored as a yes or no and summed (scale of 0–5). Household participation consisted of six separate items measuring at least weekly: cleaning of own room, cooking, general cleaning, laundry, outdoor work, and other household activities; each activity was scored as a yes or no and summed (scale of 0–6). Solitary participation consisted of two items concerning regularity of i) to read non-school books and 2) to follow the news; both items were measured on 4 item Likert scales ranging from less often/never to every day, items were summed (scale of 4–8). Social participation consisted of five items concerning regularity of: visiting friends, have friends at home, meet friends elsewhere, speak to friends over the phone, and see friends online; items were measured on 4 item Likert scales ranging from less often/never to every day and summed (scale of 4–16). A variable including other activities led by an adult leader was also included (such as music, scouts etc.) and scored as yes or no.

#### Safety and family

Perceived safety addressed perceived feeling of safety in the following areas: the classroom, on school breaks, on the way to and from school, in the neighbourhood during the day, in the neighbourhood during the night; items were scored as yes or no and summed (range 0–5). Parental monitoring consisted of one item measuring how rigorous caregivers were with knowledge of the child’s whereabouts, scored on a likert scale from 1 to 4 where 1 = very much so and 4 = not at all. Feeling part of the decision-making process at home was scored using 1 item on a range from 1 to 4 where 1 = yes always and 4 = no never. Confide in others measured whether children confided in a list of different people (parents, siblings, friends, other adults, and other) when feeling worried about something; items were scored as yes or nor and summed (range 0–6). Four items measured family relations: mother/father has time for me, and I can agree with mother/father, all scored on a 4-item scale ranging from 1 = yes always and 4 = no never.

#### Child behaviours

Digital media use consisted of hours of use of watching TV, using the computer, using social media, and playing video games; where each item was answered on a scale where 1 = < 1 h per day and 4 = > 5 h per day, items were summed (range 4–16). Risk-taking behaviours included drinking alcohol, smoking and truancy, answered as yes/no and summed (range 0–3).

#### Caregivers

General health measured on a scale where 1 = very good and 5 = very poor. Other items were answered on a yes or no scale including having a long-term disease, asthma, allergies, anxiety, physical pains, sleeping problems, using tobacco, having a disability and being the recipient of disability benefits. A sum score was created regarding caregiver adversities including physical pains, sleeping problems, tobacco use, disability (visual, mobility, hearing), allergies, asthma, long-term disease, activity benefits, and anxiety. Participation amongst the caregivers included physical exercise (indoor and outdoor as separate items), being outdoors in nature, attending cultural events such as theatre, concerts, opera and dance. Items were answered on a Likert scale ranging from never [1] to every week or more [4]. A mean score was created for individuals with a minimum of 50% of the items answered [2] as about half of the participants had one or more missing items. Finally, demographic information of the caregiver(s) was included. Caregiver in the interview as well as their partners education was included scored as 1 = Compulsory education only (finished at age 16) 2 = College (finished at age 18) 3 = Graduate education less than 3 years 4 = Graduate education 3 years or more. Whether the household could afford 12,000 SEK one month (about $1200 US) without borrowing was scored as yes or no. Financial difficulties was defined as not being able to keep up with payments and/or bills, and scored as yes or no.

### Statistical analysis

Latent class analysis (LCA) was performed in Mplus to assess whether four latent classes could be identified and supported in accordance with the two-continua model. LCA was preferred over cluster analysis as LCA (but not cluster analysis) is based on a statistical model and provides probabilities of being in the different classes.

Different class solutions were assessed (1–5 class models) and compared using the Voung-Lo-Mendell-Rubin test [58, 59] and the bootstrapped parametric likelihood ratio test [60] which compares the model with K classes to a model with K-1 classes where the bootstrap method is the most reliable. Both tests are reported using p-values where a significant test show that a K model is a better fit than a K-1 model. Other model fit indices considered include the BIC and AIC value, where the lowest value indicates the best fit [61]. The need for stable and reliable classes is also motivated using the smallest class size which should not be smaller than 5% [62]; others relax this assumption [63], however as comparisons were planned, too small groups could be an issue and a model with classes larger than 5% was preferred.

The classes were extracted and imported into STATA, where descriptive statistics concerning relevant factors and group comparisons were calculated. Statistical analysis was performed in STATA 14 and Mplus 8.7 [64]. To assess whether children with a disability were more prone to poorer mental health, children with different types of disabilities were compared across the classes. A chi-square analysis was performed to assess whether they significantly differed across classes. When assessing whether other factors differed across classes and disability, relevant analysis was performed (two-way ANOVA/Chi-square/T-test/Kruskal Wallis/Mann Whitney) (as several of the measures were on a Likert scale and not normally distributed). Results are presented by class and disability in Table 5. Kruskal Wallis is reported with ties.

### Missing data

The data for the LCA analysis (mental health problems and well-being items) were considered missing at random (MAR) based on (i) small amount of missing data (2.25%) and (ii) that the little MCAR (Missing Completely At Random) test was significant (*p* >.001) whereby MCAR was excluded. As we cannot test for MNAR (Missing Not At Random) and it is unlikely the data is MNAR, we assumed the data was MAR. Multiple imputations (five) by chained equations were conducted using MICE which assumes that data used in the imputation procedure are MAR meaning that the probability of data missing only depends on observed and not unobserved values. All variables used in the LCA analysis were imputed.

## Results

### Identifying classes using LCA

Models with different number of latent classes (1–5 classes) were compared and model fit indices are presented in Table 2. In summary, a 2-class and 4-class model were indicated to be good fits on all accounts but the BIC value; the 3-class model was not indicated to be a better fit than a 2-class model; and the 5-class model was indicated to be a good fit on all accounts but with too small class sizes. Therefore, while a 5-class solution provided the best statistical fit, it did indicate one class of small size (indicating issues in itself as well as rendering the 5-class model incapable of group comparisons across classes (same as the 3-class model)), all other classes indicated varying best statistical fit. As the aim was to assess whether the data aligned with the theoretical assumptions of a dual-continua model, we concluded that the 4-class solution did provide additional explanatory power as compared to the 2 and 5-class solution, that it provided adequate statistical fit and provided classes of sufficient size.

**Table 2:** Model fit indices LCA

	Smallest class	BIC	AIC	VLMR	LMR LRT	BPLLR
1-Class	-	112860.66	112711.62	-	-	-
2-Class	30.23% (*N* = 1111)	106585.26	106355.49	0.000	0.000	0.000
3-Class	2.75% (*N* = 101)	97309.88	96999.38	0.800	0.802	0.000
4-Class	11.5% (*N* = 423)	102374.89	95002.87	0.259	0.259	0.000
5-Class	2.74% (*N* = 101)	92891.33	92419.38	0.000	0.000	0.000

BIC = Bayesian Information Criterion, AIC = Akaike Information Criteria, VLMR = Voulg-Lo-Mendell-Rubin, p-value; LMR LRT = Lo-Mendell-Rubin adjusted LRT test, p-value; BPLLR = Bootstrapped Parametric Log Likelihood Ratio test, p-value

**Fig. 1:**
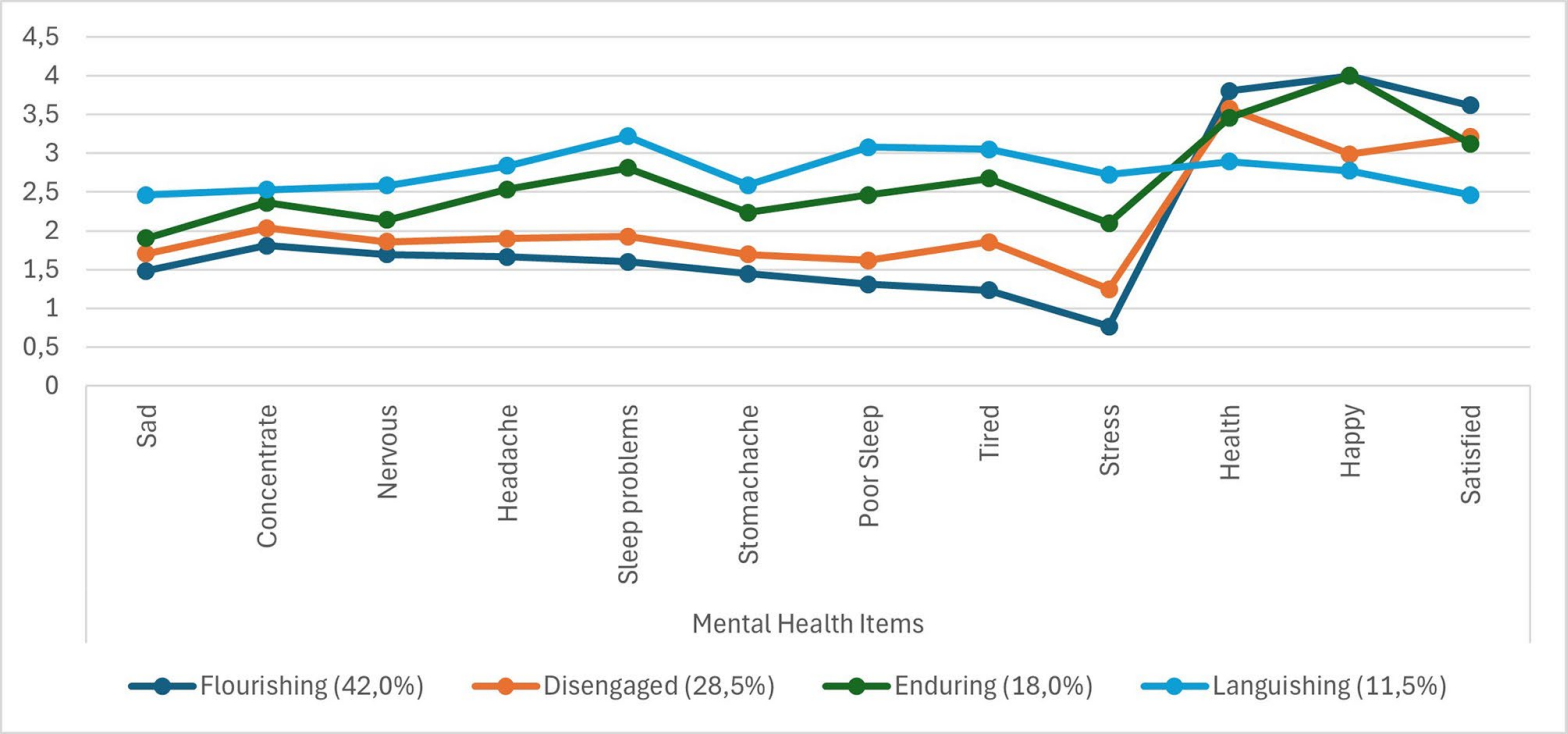
LCA 4-class solution of mental health items

The 4-class solution is presented in Fig. 1. The first 9 items in the figure are mental health problems and the last 3 items are mental health well-being. The *Flourishing* class, the dark blue line, represents a group of individuals with high mental health well-being and low mental health problems and is the largest of the classes at 42%. The *Disengaged* class, the orange line, represents a group of individuals with relatively low level of mental health problems and somewhat poor mental health well-being. The *Enduring* class, the green line, represents a group of individuals with a relatively high levels of both mental health problems and well-being. The *Languishing* class, the light blue line represents a group of individuals with high levels of mental health problems and poor well-being. Overall, the classes presented an increase in adversity (increased mental health problems and decreased well-being) with each class (from flourishing to languishing). However, consideration should be taken, as while the items for mental health problems are in line with a 4-class model, the items for well-being are not as clearly defined regarding the disengaged and enduring class. These two classes appear to only differ on the item measuring happiness within the well-being dimension, which should be considered when interpretating the results.

### Differences across classes and disability

Descriptives of gender, age and disability across classes are presented in Table 3. Children without disabilities are more prevalent in the Flourishing class, whereas children with disabilities are more prevalent in the Languishing class. In comparison to actual representation in the full sample (13.87% children with a disability), near one-quarters of the Languishing group did have a disability. A χ^2^ -test indicated significant differences between children with and without disabilities across classes (χ^2^ = 38.80, *p* <.000). There is not only an overrepresentation of children with a disability within the Languishing group; girls, independent of disability status, are overrepresented in the Languishing class, in fact, representation of girls increase with adversity of class. The result also reveals a pattern of increasing adversity with age.

**Table 3:** Descriptives of disability, gender and age across the 4 latent classes

			Females (%)	Age (Mean, SD)
Flourishing (42.0%)	No disability	88.28%	46.41	13.96 (2.24)
	Disability	11.72%	34.97	14.28 (2.04)
Disengaged (28.5%)	No disability	86.9%	52.37	14.26 (2.35)
	Disability	13.10%	41.61	14.45 (2.16)
Enduring (18.0%)	No disability	85.89%	62.64	14.94 (2.22)
	Disability	14.11%	50.55	15.18 (2.20)
Languishing (11.5%)	No disability	76.60%	74.38	15.34 (2.04)
	Disability	23.40%	74.75	15.48 (2.14)

To better understand the differences, type of disability was compared across classes (Table 4) indicating that the proportion of children with ADHD/ASD, other disabilities and comorbid disabilities differed significantly across classes and were more commonly found in the Languishing class.

**Table 4:** Disability by class, % by class

	No Disability	ADHD/ASD	Dyslexia	Hearing	Visual	Mobility	Other	Comorbid	Total
Flourishing	88.28 (1379)	1.73 (27)	4.74 (74)	1.02 (16)	1.02 (16)	0.45 (7)	1.47 (23)	1.28 (20)	100 (1562)
Disengaged	86.90 (909)	2.20 (23)	4.68 (49)	0.96 (10)	1.05 (11)	0.57 (6)	1.15 (12)	2.49 (26)	100 (1046)
Enduring	85.89 (554)	2.48 (16)	4.03 (26)	0.78 (5)	0.62 (4)	1.40 (9)	2.64 (17)	2.17 (14)	100 (645)
Languishing	76.60 (324)	5.44 (23)	4.02 (17)	0.95 (4)	0.71 (3)	0.95 (4)	5.20 (26)	5.20 (22)	100 (423)
Total	86.13 (3166)	2.42 (89)	4.52 (166)	0.95 (35)	0.92 (34)	0.71 (26)	2.12 (78)	2.23 (82)	100 (3676)
Chi2*	**38.80**, ***p*** < **.001**	**31.78**, ***p*** < **.001**	3.95, *p* =.267	1.17, *p* =.760	2.34, *p* =.504	6.45, *p* =.092	**60.10**, ***p*** < **.001**	**23.92**, ***p*** < **.001**	

*Chi2: each disability by class as to assess whether they differ across class spread

### Differences in factors related to class and disability

Factors of interest, or which were assumed likely to be associated and to possibly differ across class membership and disability/no disability, were assessed (Table 5). Overall, an increase in adverse circumstances were observed for children with a disability and for children in the more adverse classes. Children with a disability, as compared to those without, reported lower levels of participation than those without a disability across all four classes.

**Table 5:** Child and parental factors by class and disability ((M (SD) // % (n))*

							Statistical analysis to compare variable across class and disability	
		Class 1	Class 2	Class 3	Class 4	No/Dis	Class	Disability
***Participation***								
**Social participation**	No Disability	9.93 (2.47)	9.71 (2.43)	10.37 (2.14)	10.15 (2.41)	9.97 (2.41)	**F(3**,** 3675) = 9.46**, ***p*** < **.001**	**t(3674) = 4.53**, ***p*** < **.001**
	Disability	9.31 (2.76)	9.37 (2.52)	9.97 (2.55)	9.24 /(2.65)	9.44 (2.65)		
**Household participation**	No Disability	2.58 (1.33)	2.53 (1.32)	2.53 (1.30)	2.84 (1.30)	2.58 (1.32)	**F(3**,** 3673) = 4.49**, ***p*** < **.05**	t(3674) = 0.83, *p* =.408
	Disability	2.54 (1.34)	2.41 (1.31)	2.60 (1.40)	2.60 (1.37)	2.53 (1.35)		
**Cultural participation**	No Disability	2.40 (1.16)	2.41 (1.19)	2.64 (1.19)	2.57 (1.29)	2.46 (1.19)	**F(3**,** 3672) = 6.20**, ***p*** < **.001**	**t(3674) = 1.95**,** p < = 0.05**
	Disability	2.36 (1.24)	2.26 (1.18)	2.35 (1.20)	2.48 (1.34)	2.35 (1.23)		
**Physical participation**	No Disability	1.54 (0.64)	1.42 (0.69)	1.51 (0.65)	1.31 (0.71)	1.48 (0.66)	**X²(6) = 50.15**, ***p*** < **.001**	**X²(2) = 33.10**,** < 0.001**
	Disability	1.37 (0.68)	1.34 (0.71)	1.26 (0.76)	1.10 (0.74)	1.29 (0.72)		
**Stationary participation**	No Disability	5.17 (1.62)	5.25 (1.59)	5.23 (1.46)	5.50 (1.57)	5.24 (1.57)	**F(3**,**3660) = 4.53**, ***p*** < **.05**	**t(3674) = 2.57**, ***p*** < **.05**
	Disability	5.29 (1.60)	5.57 (1.64)	5.64 (1.56)	5.49 (1.62)	5.47 (1.61)		
**Other activities**	No Disability	22.77 (314)	24.45 (222)	24.37 (135)	20.99 (68)	23.35 (739)	X²(3) = 2.12, *p* =.547	X²(1) = 3.07, *p* =.080
	Disability	25.82 (47)	28.47 (39)	27.47 (25)	26.26 (26)	26.92 (137)		
***Child risk & protective factors***								
**Born in Sweden (yes/no)**	No Disability	87.96 (1213)	87.56 (796)	92.24 (511)	88.58 (287)	88.66 (2807)	**X²(3) = 7.84**,** < 0.05**	X²(1)3.81, *p* =.051
	Disability	92.89 (170)	90.51 (124)	91.21 (83)	90.90 (90)	91.57 (467)		
**Confide in others**	No Disability	2.41 (1.06)	2.20 (1.02)	2.32 (1.11)	2.21 (1.17)	2.31 (1.07)	**F(3**,** 3661) = 9.05**, ***p*** < **.001**	t(3663) = 0.57, *p* =.501
	Disability	2.37 (1.13)	2.31 (1.07)	2.26 (1.00)	2.06 (1.09)	2.28 (1.09)		
**Skip meals (yes/no)**	No Disability	37.56 (518)	47.96 (436)	61.37 (340)	78.09 (253)	48.86 (1547)	**X²(6) = 366.75**, ***p*** < **.001**	X²(1) = 0.52, *p* = 469
	Disability	33.88 (62)	51.82 (71)	60.44 (55)	70.71 (70)	50.59 (358)		
**Percieved safety**	No Disability	4.86 (0.53)	4.83 (0.54)	4.81 (0.48)	4.60 (0.82)	4.82 (0.56)	**F(3**,** 3672) = 31.37**, ***p*** < **.001**	**t(3664) = 8.06**, ***p*** < **.05**
	Disability	4.81 (0.54)	4.85 (0.45)	4.61 (0.81)	4.41 (1.03)	4.71 (0.71)		
**Have a close friend (yes/no)**	No Disability	96.14 (1320)	93.99 (845)	93.30 (515)	89.13 (287)	94.31 (2967)	**X²(3) = 57.10**, ***p*** < **.001**	**X²(1) = 15.69**, ***p*** < **.001**
	Disability	96.72 (177)	91.91 (125)	85.56 (77)	76.84 (73)	89.68 (452)		
Parental monitoring**	No Disability	1.51 (0.61)	1.61 (0.66)	1.61 (0.66)	1.68 (0.69)	1.57 (0.64)	**X²(3) = 24.59**, ***p*** < **.001**	z = 1.10, *p* =.274
	Disability	1.51 (0.61)	1.57 (0.68)	1.54 (0.56)	1.54 (0.64)	1.54 (0.63)		
Part of the decision process at home**	No Disability	1.70 (0.63)	1.80 (0.64)	1.74 (0.59)	1.87 (0.71)	1.75 (0.64)	**X²(9) = 35.52**, ***p*** < **.001**	z = 0.30, = 0.762
	Disability	1.63 (0.59)	1.80 (0.64)	1.80 (0.78)	1.96 (0.74)	1.77 (0.68)		
**Can afford 200 SEK (yes/no)**	No Disability	97.66 (1335)	95.57 (862)	98.37 (542)	94.70 (304)	96.88 (3043)	**X²(3) = 17.10**, ***p*** < **.001**	**X²(1) = 7.94**, ***p*** < **.01**
	Disability	95.03 (172)	98.48 (130)	92.13 (82)	89.80 (88)	94.40 (472)		
Mother has time for me**	No Disability	1.43 (0.60)	1.49 (0.61)	1.49 (0.58)	1.64 (0.69)	1.48 (0.61)	**X²(3) = 44.09**, ***p*** < **.001**	**z = 2.58**, ***p*** < **.01**
	Disability	1.49 (0.74)	1.55 (0.59)	1.58 (0.60)	1.71 (0.76)	1.57 (0.69)		
Father finds time for me**	No Disability	1.56 (0.75)	1.70 (0.82)	1.72 (0.79)	2.00 (1.03)	1.68 (0.82)	**X²(3) = 110.36**, ***p*** < **.001**	**z = 4.31**, ***p*** < **.001**
	Disability	1.64 (0.86)	1.93 (1.02)	1.87 (0.80)	2.21 (1.08)	1.87 (0.96)		
Agree with mother**	No Disability	1.31 (0.58)	1.49 (0.66)	1.51 (0.69)	1.75 (0.80)	1.44 (0.66)	**X²(3) = 173.67**, ***p*** < **.001**	**z = 4.42**, ***p*** < **.001**
	Disability	1.44 (0.70)	1.67 (0.70)	1.47 (0.60)	1.84 (0.94)	1.59 (0.75)		
Agree with father**	No Disability	1.35 (0.70)	1.55 (0.79)	1.52 (0.76)	1.95 (1.00	1.50 (0.79)	**X²(3) = 212.85**, ***p*** < **.001**	**z = 6.35**, ***p*** < **.001**
	Disability	1.56 (0.86)	1.83 (1.04)	1.66 (0.78)	2.08 (1.14)	1.75 (0.97)		
**Digital use**	No Disability	8.86 (2.36)	9.12 (2.41)	9.44 (2.28)	9.99 (2.08)	9.15 (2.36)	**F(3**,** 3672) = 23.85**, ***p*** < **.001**	**t(3664) = 5.68**, ***p*** < **.05**
	Disability	9.23 (2.65)	9.47 (2.53)	9.90 (2.06)	9.72 (2.25)	9.51 (2.45)		
**Problematic beahviour**	No Disability	0.16 (0.46)	0.23 (0.59)	0.44 (0.82)	0.61 (0.91)	0.27 (0.65)	**X²(3) = 78.48*p*** < **.001**	**X²(3) = 2.19**, ***p*** < **.05**
	Disability	0.14 (0.41)	0.26 (0.65)	0.48 (0.79)	0.65 (0.87)	0.33 (0.69)		
***Caregivers factors***								
General health**	No Disability	1.70 (0.78)	1.79 (0.79)	1.83 (0.83)	1.93 (0.88)	1.78 (0.81)	**X²(3) = 26.38**, ***p*** < **.001**	**Z = 4.18**, ***p*** < **.001**
	Disability	1.92 (0.77)	1.90 (0.85)	1.99 (0.94)	1.93 (0.85)	1.93 (0.84)		
**Disability benefits (yes/no)**	No Disability	9.14 (126)	9.39 (85)	9.44 (52)	12.69 (41)	9.63 (304)	X²(3) = 7.30, *p* =.063	**X²(1) = 20.69**, ***p*** < **.001**
	Disability	13.66 (25)	17.65 (24)	16.48 (15)	19.19 (19)	16.31 (83)		
**Long-term disease (yes/no)**	No Disability	27.05 (373)	31.06 (282)	28.99 (160)	35.91 (116)	29.44 (931)	**X²(3) = 13.66**, ***p*** < **.001**	**X²(1) = 15.75**, ***p*** < **.001**
	Disability	36.07 (66)	40.44 (55)	37.36 (34)	39.80 (39)	38.19 (194)		
**Asthma (yes/no)**	No Disability	8.07 (111)	7.40 (67)	7.97 (44)	9.01 (29)	7.96 (251)	X²(3) = 2.12, *p* =.546	**X²(1) = 7.48**, ***p*** < **.001**
	Disability	8.74 (16)	12.50 (17)	13.19 (12)	14.14 (14)	11.59 (59)		
**Allergi (yes/no)**	No Disability	33.55 (462)	30.21 (274)	35.57 (196)	30.60 (97)	32.65 (1029)	X²(3) = 3.82, *p* =.282	**X²(1) = 6.64**, ***p*** < **.05**
	Disability	39.01 (71)	36.76 (50)	29.67 (27)	47.96 (47)	38.46 (195)		
**Anxiety (yes/no)**	No Disability	15.41 (210)	17.59 (158)	21.05 (116)	29.28 (94)	18.45 (578)	**X²(3) = 46.16**, ***p*** < **.001**	**X²(1) = 19.35**, ***p*** < **.001**
	Disability	23.08 (42)	29.32 (39)	20.00 (18)	36.73 (36)	26.84 (135)		
**Physical pain (yes/no)**	No Disability	54.24 (748)	58.86 (525)	57.58 (319)	62.96 (204)	57.04 (1806)	**X²(3) = 8.28**, ***p*** < **.05**	**X²(1) = 13.49**, ***p*** < **.001**
	Disability	67.21 (123)	67.15 (92)	65.93 (60)	60.61 (60)	65.69 (335)		
**Sleeping problems (yes/no)**	No Disability	20.44 (279)	25.30 (229)	26.50 (146)	28.88 (93)	23.77 (747)	**X²(3) = 17.25**, ***p*** < **.001**	**X²(1) = 8.02**, ***p*** < **.05**
	Disability	27.47 (50)	32.09 (43)	30.00 (27)	29.90 (29)	29.62 (149)		
**Tobacco use (yes/no)**	No Disability	17.21 (237)	15.44 (140)	18.08 (100)	22.91 (74)	17.44 (551)	**X²(3) = 9.91**, ***p*** < **.05**	X²(1) = 3.04, *p* =.081
	Disability	20.22 (37)	19.12 (26)	21.98 (20)	22.22 (22)	20.63 (105)		
**Disability (Hearing**,** Visual**,** Mobility) (yes/no)**	No Disability	14.29 (197)	16.06 (146)	15.34 (85)	18.21 (59)	15.38 (487)	X²(3) = 3.63, *p* =.303	**X²(1) = 4.35**, ***p*** < **.05**
	Disability	17.49 (32)	18.98 (26)	24.18 (22)	17.17 (17)	19.02 (97)		
**Parental problems sum**	No Disability	2.37 (2.02)	2.52 (2.00)	2.66 (2.16)	3.03 (2.16)	2.53 (2.06)	**F(3**,** 3672) = 9.24**, ***p*** < **.001**	**t(3664) = 6.58**, ***p*** < **.001**
	Disability	3.10 (2.22)	3.21 (2.30)	3.11 (2.52)	3.37 (2.44)	3.19 (2.33)		
**Parental participation**	No Disability	2.42 (0.72)	2.40 (0.68)	2.49 (0.69)	2.32 (0.74)	2.42 (0.71)	**F(3**,** 3672) = 3.18**, ***p*** < **.05**	t(3664) = 0.19, *p* =.848
	Disability	2.43 (0.75)	2.33 (0.78)	2.49 (0.75)	2.45 (0.70)	2.42 (0.75)		
**Caregiver education**	No Disability	2.85 (1.04)	2.85 (1.01)	2.89 (0.97)	2.78 (0.96)	2.85 (1.01)	**X²(3) = 17.43**, ***p*** < **.05**	**X²(3) = 11.56**, ***p*** < **.01**
	Disability	2.68 (0.98)	2.62 (1.00)	2.71 (1.04)	2.97 (0.96)	2.73 (0.99)		
**Caregivers partners education**	No Disability	2.85 (1.00)	2.84 (0.99)	2.86 (0.96)	2.85 (1.01)	2.85 (0.99)	X²(3) = 15.42, *p* =.080	**X²(3) = 9.82**, ***p*** < **.05**
	Disability	2.69 (0.99)	2.65 (1.00)	2.54 (1.00)	2.91 (0.96)	2.69 (0.99)		
**Can’t afford 12,000 SEK**	No Disability	16.86 (230)	15.77 (141)	14.52 (80)	23.68 (76)	16.84 (527)	**X²(3) = 9.93**, ***p*** < **.05**	**X²(1) = 16.20**, ***p*** < **.001**
	Disability	21.86 (40)	25.74 (35)	28.89 (26)	22.22 (22)	24.21 (123)		
**Financial difficulties**	No Disability	2.40 (33)	4.84 (44)	3.44 (19)	4.01 (13)	3.45 (109)	**X²(3) = 11.33**, ***p*** < **.05**	X²(1) = 0.98, *p* = 323
	Disability	2.75 (5)	4.38 (6)	5.49 (5)	6.06 (6)	4.32 (22)		

* Mean and SD is presented for continous variables. Where yes/no answers are reported, the share of yes is reported in % and N is presented (by column)

** Non parametric test performed (Kruskall wallis /Mann Whitney)

### Risk and protective factors by class

Children in the flourishing class exhibit the highest level in most protective factors such as number of confidants, perceived safety and family functioning (parents having time for them, agreeing with parents, parental monitoring, and the feeling of being part of the decision-making process at home), while at the same time reporting the lowest levels in most putative risk factors, such as meal skipping, digital media use and risk-taking behaviours. Caregivers of the children in the flourishing class also experienced high levels in protective factors and low levels in putative risk factors.

On the contrary, children in the languishing class reported low levels of protective factors (such as physical participation) whilst exhibiting high levels of several putative risk factors. Children with disabilities reported the same pattern as children without a disability with one exception, digital media use. Caregivers of the children in the languishing class also reported the highest level of adversities.

The enduring and the disengaged class indicated similarities, such as averages of the flourishing and languishing classes on having a close friend and all the family functioning related factors. Differences between the classes were also observed, such as that the disengaged class generally reported lower levels than the enduring class of both positive and negative aspects, such as low levels of family functioning and participation, as well as lower levels of meal skipping, digital media use, and risk-taking behaviours. Or in other words, the disengaged class consisted of low-engagement children while the enduring featured children who face challenges but showed resilience (as indicated by high well-being). The same pattern was observed amongst children with a disability. Interestingly, children with a disability in the disengaged class reported second to worse (after the languishing class) on several of the family functioning items.

In addition, it is noteworthy that some factors seemed in line with the presence or absence of mental health well-being and problems. For example, confiding in others and being able to afford 200 SEK was higher in the classes with higher well-being and lower in the classes with mental health problems. For children with disabilities, this pattern was also observed across family functioning factors.

### Caregivers of children with a disability

Caregivers of children with a disability (as compared to caregivers of children without a disability) reported significantly higher levels of risk-taking behaviours and were more likely to report adversities such as long-term disease, generally worse health, higher anxiety, physical pains and sleeping problems. They were also more likely to receive disability support. In addition, caregivers and caregivers’ partners of children with disabilities reported significantly lower levels of education compared to caregivers of children without disabilities. This was true in all classes apart from the languishing class where, interestingly, caregivers of children with disabilities reported higher levels of education. While financial difficulties were significantly different between classes, it was not significantly different between children with and without disabilities. However, not being able to afford 12,000 SEK was more likely amongst caregivers of children with a disability.

## Discussion

This study aimed to address a research gap concerning the two-continua model and its application to children and its association with disabilities. Four findings are particularly noteworthy. *First*, concerning the feasibility of the model, results are mostly in line with the two-continua model suggesting four classes of mental health problems and well-being: flourishing, disengaged, enduring and languishing, where mental health problems increased with each respective class while well-being decreased. The results imply that the two-continua model can be applied to not only adults but also children and highlights the importance of looking at mental health problems and well-being as separate entities. *Secondly*, differences between classes in relation to other factors did support an accumulation of putative risk factors with increased levels of adversity in class and an accumulation of protective factors with decreased adversity of class, in both the child and their caregiver. In particular, social participation and physical participation act as promoting factors of well-being, highlighting the importance on focusing interventions and preventions on these two areas. *Thirdly*, the results show that having a disability is not a direct risk factor to poor mental health as indicated by the large proportion of children with a disability in the flourishing class. However, children with a ADHD/ASD, other, or comorbid disability conditions were overrepresented in the languishing class and somewhat underrepresented in the flourishing class and did report more adverse levels of putative risk and less protective factors as compared to children without a disability where the opposite pattern was observed, highlighting the importance to focus on these groups of children. In addition, the likelihood of being in an increasingly adverse class increased consecutively with increased age and being female, in line with Eriksson and Stattin’s results [51, 65], again highlighting the increased vulnerability in these groups. And *fourthly*, a much higher level of adversities for caregivers of children with disabilities were observed, irrespective of class, indicating a component of family stress related to a child’s disability, independent of the child’s mental health problems and well-being, highlighting the importance of support for caregivers of children with a disability, in line with previous literature [66].

### Class structures

Most children, independent of disability, do not report mental health problems and two out of five children were classified as *flourishing.* Amongst children with disabilities, while still overrepresented in the languishing class and underrepresented in the flourishing class, 6 out of 10 children did not report mental health problems, indicating that despite an increased risk for adversity, still more than one third were classified as flourishing. Particularly visible in this class are children with sensory disabilities or dyslexia; arguably, they may receive sufficient support to function well, due to aspects such as visibility [67, 68] or predictability [69] suggesting a disability may not be a risk factor of mental health if given sufficient support.

At the other end of the spectrum, in the *languishing* class, the differences between children with and without disabilities are larger than in any other class. Having a disability increases each putative risk factor, mirroring the results of previous studies concerning risks. For example, a study using LCA identifying risk patterns did identify an accumulation of risk factors in the least favourable class, with an over-representation of children with an ADHD/ASD diagnosis concerning parental-risk variables, as well as in the high-risk group [70].

The identification of a passive group, the *disengaged*, with neither high levels of mental health problems nor well-being, as well as lower levels of participation and *somewhat* lower levels of putative risk and protective factors, corresponds to findings of a study on preschool children [71], identifying a group with low engagement, similar to our disengaged group. This group of children may be at increased risk as low engagement impacts academic performance and happiness [72] as well as reduced supportive social relationships, as supported by the study’s results in low levels of happiness and a lack of supportive social relationships in this class. The results may be aligned with that resources in the caregiver determine how well a family manages the stress of a disability in the child and this in turn may have a major impact on the child’s well-being. This explanation is supported by our finding of high levels of caregiver adversity in this class.

### Physical participation and social relationships

The study indicates that those reporting higher levels of well-being, with or without mental health problems, also report higher levels of promoting factors, to be exact, physical participation and supportive social relationship-related factors such as higher number of confidants, high level of social participation, and higher family functioning. The results are in line with other findings such that participation is more related to well-being than psychosomatic complaints [14], that low levels of physical participation is related poor mental health [73], and that supportive relationships are one of the pillars of well-being [49]. Amongst children with a disability, similar patterns were observed but with some exceptions, for example while social participation was the highest in the enduring class, physical participation was amongst the lowest. While physical participation plays a significant role to mental health, it is possible that the present finding is a reflection on the restrictions of the disability where higher levels of restrictions decrease the possibilities of physical participation as well as being associated with poorer mental health due to the larger impact of the disability. It is also possible that the high level of well-being in this class is explained by having the resources to overcome their physical restrictions through social engagement, a notion also supported by the languishing class where children with a disability report the lowest levels of both social and physical participation. Or in other words, the present study indicates that social and physical participation are predictive of well-being, and when one of these is restricted, the other can compensate.

Overall, in classes reporting the highest participation levels and social functioning (in terms of family functioning, confidants and close friendships) higher well-being is also reported. The result suggests a strong relation between frequency of attendance in activities and social functioning as well as well-being [14, 68]. This supports Eriksson and Stattin [49] who argued that well-being should be considered an important factor rather than focusing solely on problems. Furthermore, the result of the present study supports the use of a two-continua model through highlighting that both well-being and adverse symptoms are important. Therefore, promoting children’s participation and social functioning, regardless of disability, could enhance well-being and serve as a better health facilitator than focusing solely on adverse symptoms. This may be of particular importance among children with ADHD/ASD, who face challenges with both participation and social functioning.

## Conclusion

The two-continua model provides a comprehensive framework for understanding a complex interplay between mental health, disability and participation, advocating for a holistic approach concerning children’s well-being. The study underscores the importance of promoting participation and supportive relationships to enhance well-being. In doing so, we need to focus on more than the symptoms of mental health problems. Importantly, the study highlights that it is possible to flourish even if having a disability, highlighting that different disabilities should not be viewed as one group. Differences in living circumstances probably are more important than disability status for well-being. Further studies are encouraged to investigate whether differences between disabilities lie within the symptoms of the disability itself and the support available or the implications of social construction of the disability and how it is received and handled by society.

More frequent participation, in particular physical and social participation, was found in classes with high levels of well-being, indicating that the facilitation of participation is beneficial for well-being promotion, independent of mental health problems. This can be especially central for children with disabilities with stable symptoms such as concentration difficulties and peer problems, where a treatment model focusing on mental health problems might not be as efficient.

Additionally, the study highlights resource allocation regarding the family of the child with a disability. The notion that families independent of their child’s functioning struggle, indicates a structural issue rather than a family-dependent issue. There are many calls for supporting families to improve mental health [66]. However, these are often on a relational level, and elevated stress also demands other types of support.

### Limitations

The limitations of the present study lay mainly within the measures of mental health, in particular well-being. Ideally well-being should, in line with [7], be measured using more than three items. In addition, the items used in the present study mainly concern emotional well-being and do not address social and psychological well-being comprehensively enough to be fully in line with Keyes model. Other limitations include only using items regarding frequency of participation rather than involvement or enjoyment and the study would benefit of a larger group of children with disabilities. Future studies should address this shortcoming and the results in the present study need be considered in light of this.

## Data Availability

Raw SILC/ULF and barnULF data are not available and cannot be shared openly as the data is secondary data from a third party (Swedish statistics, SCB), but restrictions apply to the public availability of these data, which were used under license for the current study, and so are not publicly available. Data must and can be applied for through SCB (https://www.scb.se/vara-tjanster/bestall-data-och-statistik/) but will need support by an ethical approval to grant access.

## Abbreviations

ADHD: Attention Deficit Hyperactivity Disorder
ASD: Autism Spectrum Disorder
barnULF: Living conditions survey of children (barn undersöknings av levnadsförhållanden)
BIC: Bayesian information criterion
BPLLR: Bootstrapped Parametric Log Likelihood Ratio test
CBCL: Child Behaviour Checklist
LCA: Latent Class Analysis
LMR LRT: Lo-Mendell-Rubin adjusted LRT test
MAR: Missing at Random
MHC-SF: Mental Health Continuum-Short Form
MICE: Multivariate imputation of chained equations
SCB: Sweden Statistics (Statistiska Centralbyrån)
SD: Standard deviation
SDQ: Strength and Difficulties Questionnaire
SILC: Statistics on Income and Living Conditions
ULF: Living conditions survey (Undersökning av levnadsförhållanden)
VLMR: Voung-Lo-Mendell-Rubin
WHO: World Health Organisation

## References

[1] World Health Organization. World Health Organization. 1948. Summary Reports on Proceedings Minutes and Final Acts of the International Health Conference held in New York from 19 June to 22 July 1946. Available from: https://apps.who.int/iris/handle/10665/85573%0AGoogle ScholarGoogle PreviewWorldCatCOPAC.

[2] Allport GW. Personality: A psychological interpretation. Holt; 1937.

[3] Jahoda M. Current concepts of positive mental health. Basic Books; 1958.

[4] Granlund M, Imms C, King G, Andersson AK, Augustine L, Brooks R, et al. Definitions and operationalization of mental health problems, wellbeing and participation constructs in children with ndd: distinctions and clarifications. Int J Environ Res Public Health. 2021;18(4):1–19. 10.3390/ijerph18041656 PMC7916140 33572339

[5] Westerhof GJ, Keyes CLM. Mental illness and mental health: the two continua model across the lifespan. J Adult Dev. 2010;17(2):110–9. 20502508 10.1007/s10804-009-9082-y PMC2866965

[6] Keyes CLM. Selecting outcomes for the sociology of mental health: issues of measurement and dimensionality. Volume 43. Source: Journal of Health and Social Behavior; 2002.

[7] Ryff CD. Psychological well-being revisited: Advances in the science and practice of eudaimonia. Psychother Psychosom [Internet]. 2014;83(1):10–28. Available from: https://psycnet.apa.org/record/2013-41405-003 10.1159/000353263 PMC4241300 24281296

[8] Söderqvist F, Larm P. Psychometric evaluation of the mental health continuum – short form in Swedish adolescents. Curr Psychol. 2023;42:2136–44.

[9] Keyes CLM. Mental illness and / or mental health?? Investigating Axioms Complete State Model Health. 2005;73(3):539–48. 10.1037/0022-006X.73.3.539 15982151

[10] Mazurek MO. Loneliness, friendship, and well-being in adults with autism spectrum disorders. Autism. 2014;18(3):223–32. 24092838 10.1177/1362361312474121

[11] Rice F, Riglin L, Lomax T, Souter E, Potter R, Smith DJ, et al. Adolescent and adult differences in major depression symptom profiles. J Affect Disord. 2019;243:175–81. 30243197 10.1016/j.jad.2018.09.015

[12] Shakya HB, Christakis NA. Association of Facebook use with compromised Well-Being: A longitudinal study. Am J Epidemiol. 2017;185(3):203–11. 28093386 10.1093/aje/kww189

[13] Twenge JM, Cooper AB, Joiner TE, Duffy ME, Binau SG. Age, period, and cohort trends in mood disorder indicators and Suicide-Related outcomes in a nationally representative dataset, 2005–2017. J Abnorm Psychol. 2019;128(3):185–99. 30869927 10.1037/abn0000410

[14] Augustine L, Lygnegård F, Granlund M. Trajectories of participation, mental health, and mental health problems in adolescents with self-reported neurodevelopmental disorders. Disabil Rehabil. 2022;44(9):1595–608. 34353177 10.1080/09638288.2021.1955304

[15] Blakemore SJ. Adolescence and mental health. The Lancet [Internet]. 2019;393(10185):2030–1. Available from: 10.1016/S0140-6736(19)31013-X 10.1016/S0140-6736(19)31013-X 31106741

[16] McGorry PD, Mei C, Dalal N, Alvarez-Jimenez M, Blakemore SJ, Browne V, et al. The lancet psychiatry commission on youth mental health. The Lancet Psychiatry. Volume 11. Elsevier Ltd; 2024. pp. 731–74. 10.1016/S2215-0366(24)00163-9 39147461

[17] Costello EJ, Mustillo S, Erkanli A, Keeler G, Angold A. Prevalence and development of psychiatric disorders in childhood and adolescence. Arch Gen Psychiatry [Internet]. 2003;60(8):837–44. Available from: http://www.ncbi.nlm.nih.gov/pubmed/12912767 10.1001/archpsyc.60.8.837 12912767

[18] Giedd JN. The teen brain: insights from neuroimaging. J Adolesc Health [Internet]. 2008 Apr [cited 2012 Jul 13];42(4):335–43. Available from: http://www.ncbi.nlm.nih.gov/pubmed/18346658 10.1016/j.jadohealth.2008.01.007 18346658

[19] Laviola G, Macrì S, Morley-Fletcher S, Adriani W. Risk-taking behavior in adolescent mice: psychobiological determinants and early epigenetic influence. Neurosci Biobehav Rev [Internet]. 2003 Jan [cited 2012 Aug 9];27(1–2):19–31. Available from: http://linkinghub.elsevier.com/retrieve/pii/S014976340300006X 10.1016/s0149-7634(03)00006-x 12732220

[20] Moore SE, Norman RE, Suetani S, Thomasc HJ, Sly PD, Scott JG. Consequences of bullying victimization in childhood and adolescence: A systematic review and meta-analysis. World J Psychiatry. 2017;7(1):60. 28401049 10.5498/wjp.v7.i1.60 PMC5371173

[21] Gorrese A. Peer attachment and youth internalizing problems: A Meta-Analysis. Child Youth Care Forum. 2016;28:177–204.

[22] Reiss F, Meyrose AK, Otto C, Lampert T, Klasen F, Ravens-Sieberer U. Socioeconomic status, stressful life situations and mental health problems in children and adolescents: results of the German BELLA cohort-study. PLoS ONE. 2019;13(3):e0213700. 10.1371/journal.pone.0213700 PMC6415852 30865713

[23] Rasic D, Hajek T, Alda M, Uher R. Risk of mental illness in offspring of parents with schizophrenia, bipolar disorder, and major depressive disorder: a meta-analysis of family high-risk studies. Schizophr Bull. 2014;40(1):28–38. 23960245 10.1093/schbul/sbt114 PMC3885302

[24] Li M, D’Arcy C, Meng X. Maltreatment in childhood substantially increases the risk of adult depression and anxiety in prospective cohort studies: systematic review, meta-analysis, and proportional attributable fractions. Psychol Med. 2016;46(4):717–30. 26708271 10.1017/S0033291715002743

[25] Eime RM, Young JA, Harvey JT, Charity MJ, Payne WR. A systematic review of the psychological and social benefits of participation in sport for children and adolescents: informing development of a conceptual model of health through sport. Int J Behav Nutr Phys Act. 2013;15(10):98. 10.1186/1479-5868-10-98 PMC3751802 23945179

[26] Wille N, Bettge S, Ravens-Sieberer U. Risk and protective factors for children’s and adolescents’ mental health: results of the BELLA study. Eur Child Adolesc Psychiatry. 2008;17(SUPPL 1):133–47. 19132313 10.1007/s00787-008-1015-y

[27] Galloway M, Conner J, Pope D. Nonacademic effects of homework in privileged, high-performing high schools. J Exp Educ. 2013;81(4):490–510.

[28] Hickman C, Marks E, Pihkala P, Clayton S, Lewandowski ER, Mayall EE et al. Young people’s voices on climate anxiety, government betrayal and moral injury: A global phenomenon. Lancet Planet Health. 2021;5(12):e863–73. 10.1016/S2542-5196(21)00278-3 10.1016/S2542-5196(21)00278-3 34895496

[29] Emerson Eric H, Chris. Mental health of children and adolescents with intellectual disabilities in Britain. Br J Psychiatry. 2007;191(DEC):493–9. 18055952 10.1192/bjp.bp.107.038729

[30] van Steensel FJA, Bögels SM, Perrin S. Anxiety disorders in children and adolescents with autistic spectrum disorders: A Meta-Analysis. Clin Child Fam Psychol Rev. 2011;14(3):302–17. 21735077 10.1007/s10567-011-0097-0 PMC3162631

[31] WHO. Children and neurodevelopmental behavioural intellectual disorders (NDBID). 2020.

[32] Coelho V, Pinto AI. The relationship between children’s developmental functioning and participation in social activities in Portuguese inclusive preschool settings. Front Educ (Lausanne). 2018;3(March) (16).

[33] Argenyi MS, Mereish EH, Watson RJ. Mental and physical health disparities among sexual and gender minority adolescents based on disability status. LGBT Health. 2023;10(2):130–7. 36301253 10.1089/lgbt.2022.0032 PMC9986008

[34] Honey A, Emerson E, Llewellyn G. The mental health of young people with disabilities: impact of social conditions. Soc Psychiatry Psychiatr Epidemiol. 2011;46(1):1–10. 19894012 10.1007/s00127-009-0161-y

[35] Berg KL, Medrano J, Acharya K, Lynch A, Msall ME. Health impact of participation for vulnerable youth with disabilities. Am J Occup Ther. 2018;72(5). 10.5014/ajot.2018.023622 10.5014/ajot.2018.023622 PMC6114191 30157012

[36] Augustine L, Bjereld Y, Turner R. The role of disability in the relationship between mental health and bullying: A focused, systematic review of longitudinal studies. Child Psychiatry Hum Dev. 2024;55(4):893–908. 36273388 10.1007/s10578-022-01457-x PMC11245418

[37] Danielsson H, Imms C, Ivarsson M, Almqvist L, Lundqvist LO, King G et al. A Systematic Review of Longitudinal Trajectories of Mental Health Problems in Children with Neurodevelopmental Disabilities [Internet]. Vol. 36, Journal of Developmental and Physical Disabilities. Springer US; 2024. 203–242 p. Available from: 10.1007/s10882-023-09914-8

[38] Antonovsky A. Unraveling the mystery of health. How people manage stress and stay well. San Francisco: Jossey-Bass; 1987.

[39] Scheier MF, Carver CS. Opti- mism, coping and health: assessment and implications of generalized out- come expectancies. Health Psycho. 1985;4:219–47. 10.1037//0278-6133.4.3.219 4029106

[40] Schwarzer R. Optimistische kompetenzerwartung: Zur erfassung einer personalen Bewa¨ltigungsres- source. Diagnostica. 1994;40:105–23.

[41] Darling N. Parenting style and its correlates. Illinois: ERIC digest EDO-PS-99- 3, clearinghouse. on Elementary and Early Childhood Education; 1999.

[42] Ezzel CE, Cupit Swenson C, Brondino MJ. The relationship of social support to physical abused children’s adjustment. Child Abuse Negle. 2000;24:641–51. 10.1016/s0145-2134(00)00123-x 10819096

[43] Lygnegård F, Granlund M, Kapetanovic S, Augustine L, Huus K. Short-term longitudinal participation trajectories related to domestic life and peer relations for adolescents with and without self-reported neurodevelopmental impairments. Heliyon [Internet]. 2021;7(4):e06784. Available from: 10.1016/j.heliyon.2021.e06784 10.1016/j.heliyon.2021.e06784 PMC8065295 33912727

[44] Di Marino E, Tremblay S, Khetani M, Anaby D. The effect of child, family and environmental factors on the participation of young children with disabilities. Disabil Health J [Internet]. 2018;11(1):36–42. Available from: 10.1016/j.dhjo.2017.05.005 10.1016/j.dhjo.2017.05.005 28624289

[45] Steinhardt F, Ullenhag A, Jahnsen R, Dolva AS. Perceived facilitators and barriers for participation in leisure activities in children with disabilities: Perspectives of children, parents and professionals. Scand J Occup Ther [Internet]. 2021;28(2):121–35. Available from: 10.1080/11038128.2019.1703037 10.1080/11038128.2019.1703037 31852318

[46] King G, Law M, Hurley P, Petrenchik T, Schwellnus H. A developmental comparison of the Out-of‐school recreation and leisure activity participation of boys and girls with and without physical disabilities. Int J Disabil Dev Educ. 2010;57(1):77–107.

[47] Simeonsson RJ, Leonardi M, Lollar D, Björck-Åkesson E, Hollenweger J, Martinuzzi A. Applying the international classification of functioning, disability, and health (ICF) to measure childhood disability. Disabil Rehabiliation. 2003;25:602–10. 10.1080/0963828031000137117 12959334

[48] Ng KW, Augustine L, Inchley J. Comparisons in screen-time behaviours among adolescents with and without long-term illnesses or disabilities: results from 2013/14 HBSC study. Int J Environ Res Public Health. 2018;15(10):2276. 10.3390/ijerph15102276 PMC6210288 30336575

[49] Kwan C, Gitimoghaddam M, Collet JP. Effects of social isolation and loneliness in children with neurodevelopmental disabilities: A scoping review. Brain Sci. 2020;10(11):1–36. 10.3390/brainsci10110786 PMC7693393 33126519

[50] Eriksson C, Stattin H. Secular trends in mental health profiles among 15-year-olds in Sweden between 2002 and 2018. Front Public Health. 2023;11. 10.3389/fpubh.2023.1015509 10.3389/fpubh.2023.1015509 PMC9978413 36875365

[51] Stattin H, Eriksson C. Person-Oriented profiles can clarify Variable-Oriented associations: the example of communication with parents and adolescents’ mental health problems. Youth. 2024;4(1):42–55.

[52] Täljedal T, Granlund M, Osman F, Norén Selinus E, Fängström K. Parenting children with disabilities in sweden: a cluster-analysis of parenting stress and sufficiency of informal and formal support. Front Psychol. 2024;15:1389995. 10.3389/fpsyg.2024.1389995 10.3389/fpsyg.2024.1389995 PMC11177875 38882520

[53] Goodman R. The Strengths and Difficulties Questionnaire: A Research Note. Journal of Child Psychology and Psychiatry [Internet]. 1997;38(5):581–6. Available from: 10.1111/j.1469-7610.1997.tb01545.x 10.1111/j.1469-7610.1997.tb01545.x 9255702

[54] Statistics Sweden (SCB). Living conditions survey of children (barnULF) [Internet]. 2024. Available from: https://www.scb.se/en/finding-statistics/statistics-by-subject-area/living-conditions/living-conditions/living-conditions-survey-of-children/

[55] Statistics Sweden (SCB). Statistics on Income and Living conditions (ULF/SILC) [Internet]. 2024. Available from: https://www.scb.se/hitta-statistik/statistik-efter-amne/levnadsforhallanden/levnadsforhallanden/undersokningarna-av-levnadsforhallanden-ulf-silc/

[56] Carlén K, Suominen S, Augustine L. The association between adolescents’ self-esteem and perceived mental well-being in Sweden in four years of follow-up. BMC Psychol. 2023;11(1):413. 10.1186/s40359-023-01450-6 10.1186/s40359-023-01450-6 PMC10676579 38007469

[57] Ivarsson M, Homman L, Danielsson H. Is the measurement of mental health problems equivalent across disability, gender, age, and cohort in children? Findings from a Swedish National survey. Scand J Public Health (in press). 10.1177/14034948251380635 41163587

[58] Lo BY, Mendell NR, Rubin DB. Testing the number of components in a normal mixture. Biometrika. 2001;88(3):767–78.

[59] Vuong QH. Likelihood ratio tests for model selection and Non-Nested hypotheses. Econometrica. 1989;57(2):307–33.

[60] McLachlan G. Finite mixture models. A wiley-interscience publication; 2000.

[61] Killian MO, Cimino AN, Weller BE. & Hyun Seo C. A systematic review of latent variable mixture modeling research in social work journals. J Evid Based Soc Work. 2019;16(2):192–210.

[62] Shanahan L, Copeland WE, Worthman CM, Erkanli A, Angold ACEJ. Sex-differentiated changes in C-reactive protein from ages 9 to 21: the contributions of BMI and physical/sexual maturation. Psychoneuroendocrinology. 2013;38(10):2209–17. 23711900 10.1016/j.psyneuen.2013.04.010 PMC3777291

[63] O’Donnell ML, Schaefer I, Varker T, Kartal D, Forbes D, Bryant RAA et al. A systematic review of person-centered approaches to investigating patterns of trauma exposure. Clin Psychol Rev [Internet]. 2017;57(September):208–25. Available from: 10.1016/j.cpr.2017.08.009 10.1016/j.cpr.2017.08.009 28919323

[64] Muthén LK, Muthén BO. Mplus user’s guide. Seventh edition. Los Angeles, CA: Muthén & Muthén; 2013.

[65] Eriksson C, Stattin H. Mental-health profiling with person-centred analysis: A study of adolescents in Sweden. Scand J Public Health. 2023;51(4):628–35. 36964644 10.1177/14034948231158850 PMC10265285

[66] FORTE. Strategisk forskningsagenda. För det nationella forskningsprogrammet om psykisk hälsa. 2023.

[67] Lingsom S. Invisible impairments: dilemmas of concealment and disclosure. Scandinavian J Disabil Res. 2008;10(1):2–16.

[68] Irwin LG, Fortune DG. Schools-based interventions for reducing stigmatization of acquired brain injury: the role of interpersonal contact and visible impairment. Arch Clin Neuropsychol. 2014;29(2):194–205. 24473119 10.1093/arclin/act118

[69] Täljedal T, Granlund M, Almqvist L, Osman F, Selinus EN, Fängström K. Patterns of mental health problems and well-being in children with disabilities in sweden: A cross-sectional survey and cluster analysis. PLoS ONE. 2023;18(7 JULY):1–18. 10.1371/journal.pone.0288815 PMC10353824 37463139

[70] Göbel K, Cohrdes C. The whole is greater than the sum of its parts: profiles of multiple mental health risk factors using latent class analysis. Child Adolesc Psychiatry Ment Health. 2021;15(1):27. 10.1186/s13034-021-00380-8 10.1186/s13034-021-00380-8 PMC8204434 34127038

[71] Åström F, Almqvist L. Patterns of observed child participation and proximity to a small group including teachers in Swedish preschool free play. Front Educ (Lausanne). 2022;7. 10.3389/feduc.2022.982837

[72] Lewis CA, Shevlin M, Francis LJ, Quigley CF. The association between church attendance and psychological health in Northern ireland: a National representative survey among adults allowing for sex differences and denominational difference. J Relig Health. 2011;50(4):986–95. 20108121 10.1007/s10943-010-9321-3

[73] Hoare E, Milton K, Foster C, Allender S. The associations between sedentary behaviour and mental health among adolescents: A systematic review. International Journal of Behavioral Nutrition and Physical Activity. Volume 13. BioMed Central Ltd.; 2016. 10.1186/s12966-016-0432-4 PMC5055671 27717387

